# Analysis of Genetic Diversity and Core Germplasm Construction of *Castanea crenata* Siebold and Zucc. Using Simple Sequence Repeat Markers and Morphological Traits

**DOI:** 10.3390/plants14131998

**Published:** 2025-06-30

**Authors:** Yanhong Cui, Xinghua Nie, Juanjuan Liu, Shihui Chu, Hanqi Liu, Kaiyuan Xu, Yi Shao, Zhannan Wang, Ruijie Zheng, Yu Xing

**Affiliations:** 1 Liaoning Institute of Economic Forestry, Liaoning Academy of Agricultural Sciences, Dalian 116031, China; cuiyh0722@163.com (Y.C.); juanjuan_9215@163.com (J.L.); 15840167311@163.com (H.L.); 13841141710@163.com (K.X.); yishao@126.com (Y.S.); wzn9060@163.com (Z.W.); 2 Beijing Key Laboratory for Agricultural Application and New Technique, College of Plant Science and Technology, Beijing University of Agriculture, Beijing 102206, China; niexinghuabua@163.com (X.N.); shihuichul@163.com (S.C.)

**Keywords:** *Castanea crenata*, SSR, genetic diversity, core collection, germplasm management

## Abstract

This study investigates the taxonomic status, phylogenetic relationships, and genetic diversity of Japanese chestnut (*Castanea crenata* Siebold & Zucc.) in Liaodong, China, and across East Asia. Additionally, it evaluates core germplasm resources through cluster and population structure analyses using simple sequence repeat (SSR) marker data from 13 *Castanea henryi*, *18 Castanea seguinii*, and 27 *Castanea mollissima*, and 142 Japanese chestnut resources. The results show that the East Asian *Castanea* genus forms a monophyletic group with distinct interspecific boundaries. Japanese chestnut and two varieties/lines of *C. seguinii* (187 and 170) form a sister clade, indicating a close phylogenetic relationship. All Japanese chestnut resources are divided into two branches, with considerable admixture. The genetic diversity analysis revealed that the 142 Japanese chestnut varieties/lines collectively possessed 141 allelic loci, with genetic distances (GDs) ranging from 0.429 to 0.880 with an average of 0.740. Based on unique characteristics, seven resources with distinctive features were selected as mandatory. A total of 41 core germplasm resources were finally determined using the simulated annealing method. The comparative analysis revealed that, aside from a notable difference in polymorphic information loci, the core germplasm and original germplasm showed no significant differences in other genetic diversity parameters. This indicates that the 41 core germplasm resources effectively preserve the genetic diversity of the original germplasm and have been influenced by artificial selection. This study provides a scientific basis for conserving and using *C. crenata* germplasm resources.

## 1. Introduction

Chestnut, a nutrient-rich and highly valued dried fruit crop has become a globally significant economic crop because of its high nutritional content, low fat levels, and abundance of riboflavin and various vitamins [1,2]. Its unique edible and medicinal value have driven widespread cultivation and consumption worldwide. The literature indicates that the genus *Castanea* comprises seven main species, *Castanea henryi* (Skan) Rehder and E.H.Wilson, *Castanea mollissima* Blume, *Castanea seguinii* Dode, *Castanea sativa* Mill., *Castanea crenata*, *Castanea dentata* (Marshall) Borkh., and *Castanea pumila* (L.) Mill., that exhibit diverse geographical distributions, growth characteristics, and economic values [3,4]. Notably, *C. crenata*, primarily distributed in Japan, the Republic of Korea, North Korea, and the Chinese provinces of Liaoning and Shandong, stands out among the *Castanea* species globally. It is characterized by large nuts, strong adaptability, cold tolerance, and high productivity [5,6]. In addition to these favorable traits, Japanese chestnut shows robust resistance to various diseases, serving as a key source of stress resistance genes for *Castanea* species worldwide [7]. Recent advances in breeding techniques have enabled the cultivation of numerous hybrid varieties/lines with strong resistance and exceptional productivity, marking significant progress in developing the chestnut industry [8].

Genetic diversity, the cornerstone of biodiversity, is crucial for maintaining species stability and driving their evolution. In genetic diversity research, morphological, cytological, and molecular marker techniques are widely applied. Morphological methods are intuitive and straightforward and can reveal genetic variation and assist in identifying potential target traits [9]. With technological advancements, molecular marker techniques have become vital for core germplasm construction in crops, advancing resource conservation, innovative utilization, and breeding [10,11]. Simple sequence repeat (SSR) molecular marker technology, known for its stability, polymorphism, and ease of operation, is the standard for core germplasm bank construction and is endorsed by the International Union for the Protection of New Varieties/Lines of Plants (UPOV) for plant DNA fingerprinting, providing a solid basis for subsequent research [12,13,14,15]. A plethora of researchers have used SSR molecular markers for germplasm resource screening. For example, Kang et al. analyzed Guangxi litchi germplasm and established a core germplasm bank [16]. Li et al. combined phenotypic traits with SSR markers to construct a core germplasm system for Chinese fuzzy kiwifruit [17]. In chestnut research, Inoue et al. first reported the development of *Castanea* SSR markers, achieving effective cross-species amplification in Japanese and European chestnuts with 17 *Castanea* SSR primer pairs. These primers showed high genetic diversity in three *Castanea* species (Chinese, Japanese, and European chestnuts) [18]. Nie et al. screened 330 SSR markers and identified 18 highly polymorphic primers to analyze the genetic diversity of 146 Chinese chestnut varieties [12]. Jiang et al. analyzed the genetic structure of 95 Chinese chestnut varieties using 41 SSR loci and classified them into three groups [19]. Pereira-Lorenzo et al. analyzed the population structure of 132 European chestnut varieties with 24 highly polymorphic SSR markers, identifying two main groups corresponding to Spanish and Italian varieties, and observed high genetic diversity [20]. Zulfiqar et al. analyzed 252 *C. mollissima* samples across 11 locations via SSR markers and determined that the Qinling–Daba Mountain population has the highest genetic diversity, with 89% of genetic variation existing within individuals/populations. Moreover, they identified five genetic clusters, offering key insights for Chinese chestnut conservation and utilization [21]. Liu et al. used fluorescence SSR markers to construct the core germplasm for national chestnut varieties/lines, supporting new variety creation and resource conservation [10]. Nishio et al. conducted association analyses on *C. crenata* Siebold and Zucc. (Japanese chestnut) resources using SSR and single nucleotide polymorphism (SNP) technologies, identifying trait-linked markers and assessing genetic diversity and kinship for hybrid breeding potential [5,6].

In China, especially in the northeastern provinces, Japanese chestnut cultivation has achieved remarkable success and has become a vital pillar industry in the mountainous regions of Liaoning Province [22]. However, with an expansion in cultivation scale and diversification of varieties/lines, the issue of confusion in Japanese chestnut germplasm resources has become increasingly prominent [22,23]. The historical introduction of Chinese chestnut (*C. mollissima*) and Japanese chestnut into each other’s territories, coupled with overlapping ecological regions, has led to unclear pedigrees and classifications in variety selection and breeding, further exacerbating the confusion in germplasm resources [24]. This confusion not only affects the genetic purity of Japanese chestnut but also hinders the promotion and application of superior varieties/lines, posing a potential threat to sustainable development in the chestnut industry. Considering the abundance and diversity of plant resources, along with the high costs associated with comprehensive preservation, establishing a core germplasm bank has become a crucial strategy for conserving and efficiently utilizing germplasm resources [10]. This strategy involves scientifically selecting the minimum number of germplasm resources required to comprehensively represent the genetic diversity of the entire population, thus maximizing the coverage of genetic information while reducing redundancy, enhancing management efficiency, and improving resource utilization [14].

Therefore, resolving the ambiguity in Japanese chestnut germplasm resources by conducting core germplasm screening is particularly important. Employing scientific and effective methods to select core germplasm with superior traits, high genetic value, and broad adaptability from a vast number of germplasm resources is of great significance for conserving and utilizing Japanese chestnut germplasm resources and promoting the healthy development of the chestnut industry. This study aims to comprehensively utilize SSR molecular marker technology to screen and identify core Japanese chestnut germplasm resources, elucidate its genetic diversity and genetic structure, clarify the genetic relationships and breeding pedigrees among varieties/lines, and provide robust support for subsequent genetic breeding and variety improvement. The ultimate goal is to contribute wisdom and strength to the sustainable development of the chestnut industry in China and even globally.

## 2. Results and Analysis

### 2.1. Cluster Analysis of the Original Chestnut Germplasm (Varieties and Lines)

**Figure 1:**
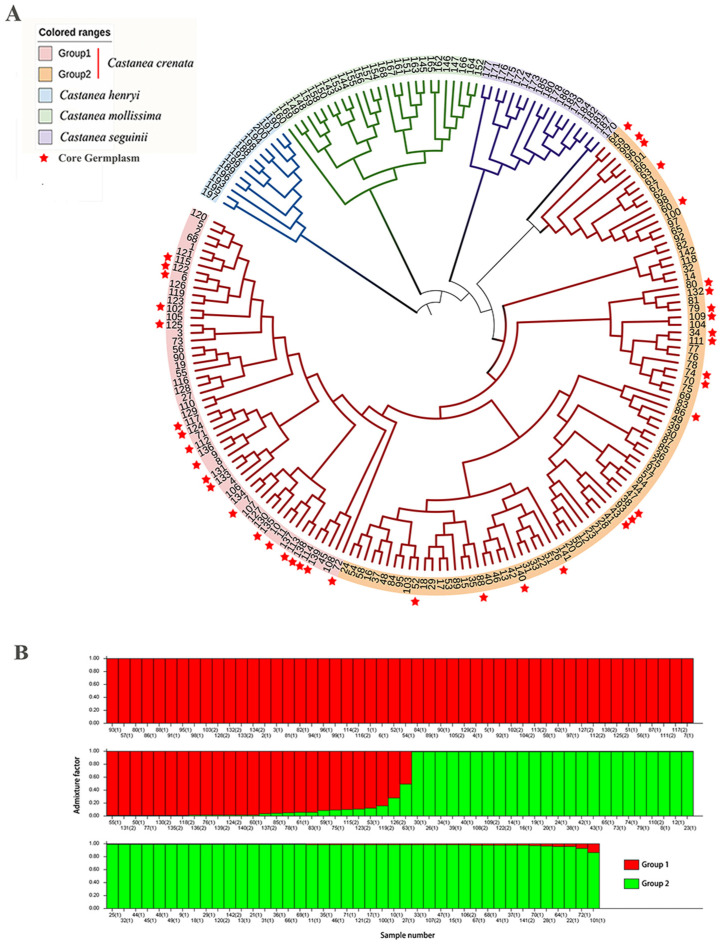
UPGMA dendrogram (**A**) and group structure (**B**) of all Japanese chestnut germplasm. (1) represents domestic Pop. 1 and (2) represents foreign Pop. 2 in Panel (**B**).

To clarify the taxonomic status of Japanese chestnut in Northeast China and other East Asian countries, as well as its phylogenetic relationships with other *Castanea* species, this study included 142 *C. crenata* (samples No. 1–142), 27 *C. mollissima* (samples No. 142–169), 18 *C. seguinii* (samples No. 170–180, and 13 *C. henryi* (samples No. 188–200), and resources for cluster and population structure analyses. The results (Figure 1A) indicate that branch lengths reflect genetic variation and distance between materials, with shorter branches signifying closer genetic relationships. The entire East Asian *Castanea* genus forms a monophyletic group with distinct interspecific boundaries. *C. henryi* is positioned at the base of all resources, while Chinese chestnut and 16 varieties/lines of *C. seguinii* are located at the sub-base. Japanese chestnut and two varieties/lines of Chinese *C. seguinii* (187 and 170) form a sister clade, indicating a close phylogenetic relationship. The cluster analysis of Japanese chestnut varieties/lines and lines only (Figure 1A) shows that all resources are primarily divided into two branches. Group 1 mainly consists of foreign varieties/lines mixed with 20 local Japanese chestnut varieties and lines from China, including Resources 1–5. Group 2 is dominated by domestic Japanese chestnut varieties/lines, including eight Japanese chestnut varieties/lines and lines (111, 148, 144, etc.) originally from foreign countries. The population structure analysis further confirms that all Japanese chestnut resources are roughly divided into two parts; however, considerable admixture exists. An admixture coefficient exceeding 0.8 in the population structure is considered indicative of significant gene flow (Nm) (Figure 1B).

### 2.2. Genetic Diversity of the Japanese Chestnut Germplasm Resources

The analysis of genetic diversity across all resources revealed that the 142 Japanese chestnut varieties/lines collectively possessed 141 allelic loci. The genetic distance (GD) ranged from 0.429 to 0.880, with an average of 0.740. The number of alleles per primer pair (Na) varied from 4 to 12, averaging 8.294. The effective number of alleles (Ne) fell between 1.751 and 8.313, with a mean of 4.423. The observed heterozygosity (Ho) spanned from 0.380 to 0.838, averaging 0.640, while the expected heterozygosity (He) ranged from 0.429 to 0.880, with a mean of 0.740. The polymorphism information content (PIC) ranged between 0.386 and 0.868, averaging 0.705. The Shannon index (*I*) ranged from 0.820 to 2.236, with an average of 1.625. These data indicate that the selected SSR markers display rich polymorphism, and the 142 Japanese chestnut varieties/lines in this study exhibit high heterozygosity and substantial genetic diversity (Table 1).

To elucidate the total genetic variation among Japanese chestnut populations both domestically and internationally, as well as the distribution of this variation within and between populations, a molecular variance analysis (AMOVA) was performed on the two distinct populations of Japanese chestnut in this study (Table 2). The results revealed that the majority of the genetic variation was partitioned within populations, accounting for 93% of the total variation, whereas variation between populations constituted only 7%. Furthermore, the Nm among *Castanea* populations was estimated at 3.581, exceeding 1. This high Nm indicates frequent genetic exchange between populations, in turn mitigating the degree of genetic differentiation among populations that might otherwise arise from genetic drift.

### 2.3. Selection of the Core Germplasm Resources

**Figure 2:**
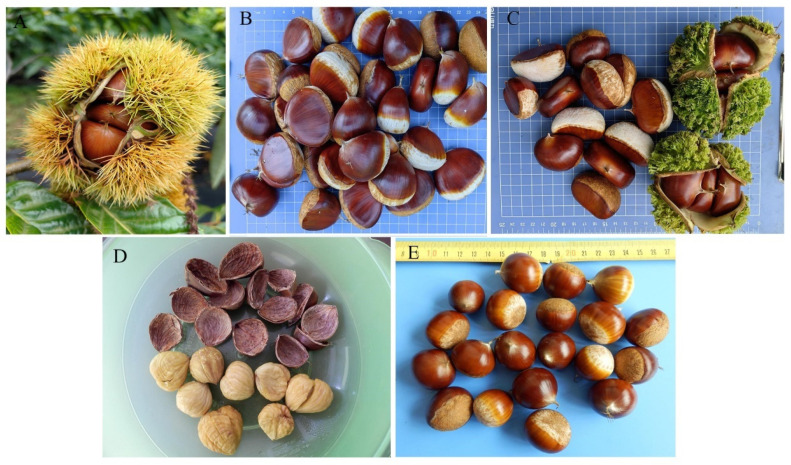
Unique germplasm resources. (**A**) Early maturing type: Liaoli Zaofeng; (**B**) giant chestnut type: 1601; (**C**) extremely short-spine type: Wuci; (**D**) easily detachable tannin skin type: Nonglin No. 8; and (**E**) single-fruit type: 08-3.

Based on unique characteristics, such as maturity period, spine length, chestnut shape, chestnut size, and degree of tannin skin detachment, seven samples with distinctive features were selected from the entire collection. These seven samples were designated as core genetic resources. These resources are expected to play a crucial role in future resource utilization, germplasm creation, and genetic research. Specifically, the early maturing germplasm is Liaoli Zaofeng (Figure 2A), which matures at the end of August, which is 5–45 days earlier than other Japanese chestnut resources. The large-grained germplasm, 1601 (Figure 2B), has an average single-nut weight of 28.03 g, approximately 7.02–8.50 g higher than other varieties/lines. The extremely short-spined germplasm is Wuci (Figure 2C), with a spine length of approximately 6 mm, 14–18 mm shorter than other varieties/lines. The germplasm with easily detachable tannin skin is Nonglin No. 8 (Figure 2D), whose tannin skin can be completely removed, unlike other Japanese chestnut resources. The single-grain germplasms, 08-3 (Figure 2E) and Hongqi Duguo, differ from the majority of Japanese chestnut resources, as each bur contains only one nut instead of the usual three.

### 2.4. Analysis of the Genetic Diversity in Core Germplasm

**Figure 3:**
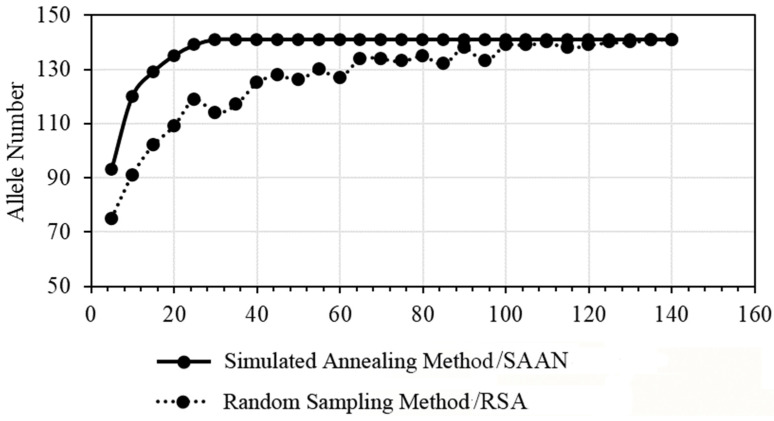
Comparison of the results of two core germplasm construction models.

As shown in Figure 3, when constructing the core germplasm using the simulated annealing method, the number of detected loci tends to plateau and performs better compared with the random sampling method, especially when considering 30 resources. Consequently, the simulated annealing-based sampling method was selected to construct the core germplasm of Japanese chestnuts. To better represent the entire range of Japanese chestnut resources, those with superior traits, distinctive characteristics, and the primary cultivated varieties/lines were included as core genetic resources. After comprehensive consideration, a total of 41 core germplasm resources were identified.

### 2.5. Validation of the Core Germplasm Rationality

**Figure 4:**
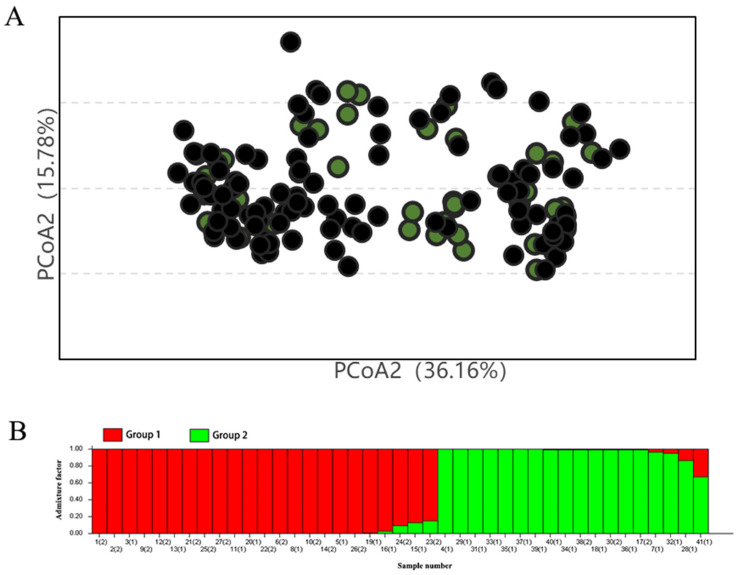
PCA distribution and group structure of the core germplasm. In Panel (**A**), the core germplasm taxa are shown in green. Black represents all germplasm accessions. In Panel (**B**), (1) represents domestic resources, and (2) represents foreign resources.

To verify the rationality, reliability, and scientific basis of the 41 core germplasm resources, first, a principal coordinate analysis (PCoA) was performed on their distribution within the entire resource pool, in addition to a population structure analysis of the core germplasm. Figure 4 shows that the tangerine-colored portion represents the core germplasm, while the black portion represents the remaining germplasm after screening. Principal coordinates 1 and 2 account for 36.16% and 15.78% of the variation in locus information data, respectively. This indicates a relatively even distribution of the core germplasm within the entire germplasm resource pool, thus confirming the rationality of the screening results. The population structure analysis reveals that the 41 core germplasm resources, similar to the entire germplasm resource pool, can be broadly divided into two groups. The population structure analysis indicates that the 41 core germplasm samples are representative of the entire germplasm collection and are broadly divided into two major groups.

### 2.6. Genetic Diversity Parameters Comparison

A comparative analysis of the genetic diversity parameters was conducted between the 41 core germplasm resources and the original 142 germplasm resources. The results showed a retention rate of 28.87% for the core germplasm. The core germplasm retained 100% of the allelic loci (141) identified in the original germplasm (Table 3). The GD in the core germplasm ranged from 0.438 to 0.888, with an average of 0.727. Na and Ne ranged from 2.00 to 11.00, with average values of 0.794 and 4.236, respectively, and retention rates of 87.94% and 95.77%. Ho and He ranged from 0.366 to 0.780 and 0.438 to 0.888, averaging 0.633 and 0.727, with retention rates of 98.44% and 98.65%. The minor allele frequency (MAF) ranged from 0.159 to 0.716, with an average of 0.405 and a retention rate of 104.92%. PIC ranged from 0.387 to 0.877, with an average of 0.69 and a retention rate of 97.18% (Table 4). Statistical analysis via *t*-test indicated that, except for a significant difference in polymorphic information loci, no significant differences were observed in other genetic diversity parameters between the core and original germplasm, indicating that the 41 core germplasm resources effectively preserve the genetic diversity of the original germplasm and suggest that these resources have been influenced by artificial selection (Table 5).

## 3. Discussion

### 3.1. Analysis of the Genetic Diversity in Japanese Chestnut

Using SSR markers for population genetic diversity studies is a highly convenient and efficient method, a conclusion validated in numerous prior studies [16,17]. The polymorphism, stability, and reproducibility of SSR molecular markers are crucial for the success of genetic research [10,11]. To improve genotyping accuracy and effectiveness in this study, 17 high-performance SSR primer pairs were screened and used for subsequent experiments and analyses based on Nie et al.’s research [12]. The results showed that these 17 SSR primer pairs detected a total of 141 allelic loci, with an average of 8.29 per marker. This is higher than previously reported SSR loci in *Castanea* species, such as in Müller et al. [25], Jiang et al. [19], and Terakami et al. [26]. This indicates the effectiveness and high resolution of the SSR primers used, enabling a more accurate assessment of the genetic characteristics of Japanese chestnut varieties/lines.

The PIC is a key metric for assessing SSR locus polymorphism. In this study, PIC values across loci ranged from 0.386 to 0.868, averaging 0.705. These highly polymorphic SSRs are significant for revealing genetic differences among Japanese chestnut varieties/lines. The *Ho* of the 142 Japanese chestnut varieties/lines ranged from 0.380 to 0.838, averaging 0.640, aligning with Nishio et al.’s calculations [5,6] and reflecting the typical high heterozygosity of *Castanea* species resulting from their self-incompatibility and reliance on regional hybridization for reproduction. Moreover, Japanese chestnut’s *Ho* is slightly higher than that of Chinese chestnut (0.652), confirming frequent hybridization among Japanese chestnut varieties/lines. In addition, the population structure analysis revealed genetic components from different subpopulations within Japanese chestnut varieties/lines, indicating complex genetic relationships. This complexity is important for understanding their genetic diversity and evolutionary history and provides valuable insights for Japanese chestnut variety improvement and genetic breeding.

This study further explores the taxonomic position of Japanese chestnut within the genus *Castanea* in Liaodong, China, and other East Asian countries, as well as its genetic relationships with other *Castanea* species, through clustering and population structure analysis. The results clearly demonstrate the monophyletic character of East Asian *Castanea* species and the significance of interspecific boundaries, providing an important basis for further elucidating the phylogeny of this genus. Notably, cone chestnut occupies a basal position among all resources, indicating its ancient status on the evolutionary tree. In contrast, Chinese chestnut and the majority of thorny chestnut varieties/lines are located at the sub-basal positions. This finding is generally consistent with the research results of Nie et al. (2022), providing new information for understanding the evolutionary history of these species [17]. Notably, the sister-group relationship formed between Japanese chestnut and two specific varieties/lines of *C. seguinii* (187 and 170) strongly indicates a close genetic relationship between Japanese chestnut and *C. seguinii*. This discovery not only enriches our understanding of the genetic relationships among *Castanea* species but also provides potential hybrid parent combinations for future genetic improvement and germplasm resource utilization [6].

In the separate clustering analysis of Japanese chestnut varieties/lines, a clear differentiation trend was observed between domestic and foreign varieties/lines, accompanied by significant admixture. Group 1 is dominated by foreign varieties/lines but includes a certain number of native Japanese chestnut varieties/lines in China, potentially reflecting historical exchanges and introductions among varieties/lines. In contrast, Group 2 is mainly composed of domestic varieties/lines but also accommodates certain foreign-introduced varieties/lines, indicating extensive Nm and variety fusion in the cultivation history of Japanese chestnut. This admixture is further confirmed in the population structure analysis, where results with admixture coefficients exceeding 0.8 indicate frequent gene exchanges among Japanese chestnut resources. In addition, this finding is consistent with the research results of Nie et al. [6], which is of great significance for maintaining and utilizing genetic diversity [6]. Future research can further focus on the mechanisms of gene exchange among these closely related species and how to harness this genetic diversity to cultivate new varieties/lines of *Castanea* with high yield, stress resistance, and superior quality.

### 3.2. Analysis of the Core Germplasm Construction for Japanese Chestnut

The collection and preservation of germplasm resources are crucial for conserving species’ genetic diversity and promoting breeding innovation. However, faced with abundant germplasm resources, efficiently conducting genetic and breeding research poses a significant challenge [27]. Therefore, constructing a core germplasm bank involving scientifically screening and concentrating high-quality germplasm resources holds profound significance for enhancing resource utilization efficiency and value [28,29]. In this process, the selection of a construction strategy is particularly critical as it directly determines the quality of the selected core germplasm samples. Additionally, NA, serving as an important measure of genetic diversity [30], significantly impacts breeding effectiveness [31]. Liu et al. used 342 Chinese chestnut varieties/lines as materials and amplified them with 21 pairs of SSR fluorescent primers, constructing 85 core germplasm banks based on stratified sampling, a simulated annealing algorithm, and a random search algorithm [10]. To further optimize the representativeness and reliability of the core germplasm, this study referenced previous research on Chinese chestnut, attempting to construct core germplasm banks using the simulated annealing method and random sampling method and subsequently comparing the differences in allele number retention between these strategies. The aim was to use the most streamlined germplasm resources to maximize the reflection of the genetic diversity characteristics of the original germplasm. The results determined the simulated annealing method to be the optimal strategy for constructing core germplasm resources. Ultimately, this study screened and identified 41 germplasm resources as members of the core germplasm bank, representing approximately 28.87% of the germplasm bank. These resources comprehensively and adequately reflect the genetic diversity characteristics of the original germplasm. This proportion is consistent with the range of core germplasm sampling proportions (5% to 40%) proposed in previous studies [32], validating the rationality and effectiveness of the research method. The simulated annealing algorithm based on allele number can represent the genetic diversity of the entire original population with minimal resources and improve the preservation of genetic diversity.

According to Zhao et al., polymorphism at loci can be considered high, medium, or low value when the GD is >0.5, between 0.5 and >0.25, or <0.25, respectively [29]. In this study, the average GD values for 142 Japanese chestnut varieties/lines and the selected 41 core germplasm accessions across 17 SSR markers were 0.740 and 0.727, respectively, with PIC values of 0.71 and 0.69, both exceeding 0.5. These results strongly demonstrate that the 142 Japanese chestnut varieties/lines in this study possess relatively high genetic diversity. Meanwhile, the screened core germplasm accessions also exhibit high polymorphism, ensuring their representativeness and importance in genetic studies.

In summary, through in-depth analysis of the genetic diversity parameters between the core germplasm and the original germplasm, based on 142 samples of Japanese chestnut and a total of 200 samples across all species, this study confirms that the selected core germplasm exhibits extremely high efficiency in retaining genetic information. This finding provides valuable reference information for variety improvement and genetic breeding of Japanese chestnut and also offers useful insights for the screening and construction of core germplasm in other crops.

## 4. Materials and Methods

### 4.1. Plant Materials and Experimental Site Conditions

The experimental materials in this study were collected from 2022 to 2024, comprising a total of 200 samples, with detailed information presented in Table S1. These samples consist of 142 *C. crenata* (samples No. 1–142), 27 *C. mollissima* (samples No. 142–169), 18 *C. seguinii* (samples No. 170–180, and 13 *C. henryi* (samples No. 188–200) samples. Ultimately, the Japanese chestnut varieties/lines were sourced from the Japanese Chestnut Resource Garden of Liaoning Province Economic Forest Research Institute, specifically from the Chestnut Germplasm Resource Nursery located at Songmudao Experimental Base. The 142 Japanese chestnut varieties/lines mainly originate from Japan, North Korea, the Republic of Korea, and China. Within China, they are primarily found in Dalian and Dandong cities of Liaoning province and Shandong province. The nursery is situated in Songmudao Village, Puwan New District, Dalian City (121°45′57.348″ E, 39°24′19.163″ N). The terrain in this area is higher in the north and lower in the south. It has a temperate continental monsoon climate with distinct seasons: mild winters, early springs, hot summers, and cool autumns. The region is well-endowed with rainfall and sunshine. The soil is weakly acidic brown earth, which is beneficial for growing Japanese chestnuts. For each sample, 6–8 young leaves were collected, frozen in liquid nitrogen, and then stored in a low-temperature refrigerator at −20 °C.

### 4.2. Experimental Methods

#### 4.2.1. DNA Extraction

Young leaves from various Japanese chestnut varieties/lines were gathered for DNA extraction. Each sample was weighed to 0.1 g. Using an efficient plant genomic DNA extraction kit (DP350) from Tiangen Biotech Co., Ltd. (Beijing, China), DNA was extracted from all samples. A spectrophotometer (Thermo NanoDrop 2000, Waltham, MA, USA) was used to assess the concentration and quality of the extracted DNA, and 1% agarose gel electrophoresis was used to verify the DNA’s integrity. Qualified DNA samples were stored in a −20 °C refrigerator.

#### 4.2.2. Fluorescent Capillary Electrophoresis SSR-PCR

In this study, 17 specific SSR primer pairs were used. These primers were previously screened and identified by the Molecular Developmental Biology Laboratory of Chinese Chestnut at the Beijing University of Agriculture and are detailed in Table 2 [4]. TsingKe Biotechnology Co., Ltd. (Beijing, China) provided the primer synthesis service. The polymerase chain reaction (PCR) amplification system was prepared at 20 μL, containing 1 μL of template DNA (20~50 ng·μL^−1^), 10 μL of 2 × Taq PCR Master Mix (Takara, Dalian, China), 0.1 μL of forward primer (10 μM), 0.3 μL of reverse primer (10 μM), 0.2 μL of M13 primer (10 μM) with fluorescent labels (FAM, HEX, and ROX) (TsingKe, Tianjin, China), and 9.4 μL of ddH_2_O. The reaction protocol was as follows: initial denaturation at 94 °C for 3 min, followed by 35 cycles of denaturation at 94 °C for 30 s, annealing at 56 °C for 30 s, and extension at 72 °C for 30 s, with a final extension at 72 °C for 10 min and storage at 10 °C. The PCR amplification was performed using a BIO-RAD PCR Thermal Cycler T100 (Waltham, MA, USA).

The PCR amplification products of the 17 fluorescent SSR primer pairs were analyzed using capillary electrophoresis with an ABI 3730XL DNA sequencer (Applied Biosystems, Foster City, CA, USA) to obtain fragment position information in the mixture. Gene Marker V 2.2.0 software (Soft Genetics LLC, State College, PA, USA) was used to read the specific fragment size information of the alleles.

#### 4.2.3. Data Analysis

Raw SSR data in the “bp value” format were converted into PowerMarker format in an Excel spreadsheet, following the specified format requirements of the Power-Marker V3.25 software [33]. Missing data were assigned a value of “9”. Genetic diversity indices, including GD, Na, Ne, Ho, He, I, minor allele frequency (MAF), and PIC, were calculated using Power-Marker V3.25 software and GenAlEx 6.51 [34,35]. Nei’s GD among resources was assessed, and a UPGMA clustering tree was constructed using FigTree v1.4.3. Population structure analysis was performed using Structure 2.3.4 [36]. PCoA and AMOVA were carried out on the materials with GenAlEx 6.51, and parameters such as the Nm fixation index of genetic differentiation (Fs) between populations were calculated.

#### 4.2.4. Construction of the Core Germplasm

(1) Selection of the Core Germplasm Based on a Simulated Annealing Algorithm

To establish an appropriate core germplasm, a simulated annealing algorithm based on the maximum number of alleles (SAAN) was employed to screen and construct the core germplasm of Japanese chestnut varieties/lines. In the PowerMarker v3.25 software, a gradient sample selection scheme was established to calculate the number of allelic loci retained in samples of different capacities, thus determining the final number of core germplasm samples to be selected. To ensure the retention of as many allelic loci as possible, a gradient selection scheme with the following numbers was adopted from 5 to 140 in steps of 5. This scheme was used to calculate the number of allelic loci for different sampling sizes, allowing for the determination of the optimal selection number when the allelic loci were maximized.

(2) Selection of the Core Germplasm Based on a Random Search Algorithm

To construct the core germplasm of chestnut varieties/lines, a random search algorithm based on the maximum Na (random search algorithm, RSA) was utilized. In addition, this method employed the aforementioned gradient selection scheme based on the simulated annealing algorithm, comparing the number of allelic loci for different sampling sizes to determine the optimal selection number. The sampling sizes and numbers of allelic loci obtained from the strategies mentioned above were compared to finalize the sampling size, which in turn was used to evaluate the optimal strategy among the core germplasm construction strategies. Finally, *t*-tests were conducted using Prism software [33] to compare the Na, Ne, Ho, He, and PIC values between the core germplasm and the original germplasm, thus verifying the accuracy, scientific validity, and reliability of the constructed core germplasm.

## 5. Conclusions

The East Asian *Castanea* genus forms a monophyletic group with clear interspecific boundaries, and *C. henryi* is positioned at the base of all resources. Japanese chestnut and two varieties/lines of Chinese *C. seguinii* form a sister clade, indicating a close relationship. Japanese chestnut varieties/lines are primarily divided into two branches, one mainly consisting of foreign varieties/lines and the other dominated by domestic ones, but with considerable admixture, indicating significant Nm. Japanese chestnut exhibits rich genetic diversity and high heterozygosity. The majority of genetic variation exists within populations resulting from frequent Nm, reducing genetic differentiation that might arise from genetic drift. This study selected samples with unique characteristics, such as early maturation, large grain size, extremely short spines, easy peeling of the tannin skin, and single grains, as core genetic resources for future utilization, germplasm creation, and genetic research. Using the simulated annealing method to construct core germplasm resources, 41 core germplasm resources were identified as representatives of the genetic diversity of Japanese chestnut. Compared with the original germplasm, the core germplasm shows high retention rates of genetic diversity parameters, preserves almost all allelic loci, and displays no significant differences in genetic diversity parameters. This indicates that the core germplasm effectively preserves the original genetic diversity and has been influenced by artificial selection.

## Figures and Tables

**Table 1:** Genetic diversity statistics of 142 Japanese chestnut resources.

Locus	GD	MAF	Na	Ne	*I*	Ho	He	*F*	PIC
CmSI0509	0.800	0.254	6.000	4.998	1.684	0.687	0.800	0.142	0.770
CmSI0561	0.429	0.729	5.000	1.751	0.820	0.380	0.429	0.114	0.386
CmSI0614	0.741	0.445	10.000	3.860	1.676	0.593	0.741	0.199	0.714
CmSI0658	0.768	0.410	11.000	4.314	1.813	0.629	0.768	0.181	0.744
CmSI0735	0.656	0.479	4.000	2.905	1.199	0.681	0.656	−0.038	0.598
CmSI0742	0.719	0.423	7.000	3.557	1.426	0.458	0.719	0.363	0.676
CmSI0853	0.805	0.320	9.000	5.134	1.829	0.755	0.805	0.062	0.781
CmSI0871	0.862	0.234	12.000	7.270	2.162	0.660	0.862	0.235	0.848
CmSI0883	0.736	0.441	10.000	3.787	1.658	0.627	0.736	0.147	0.706
CmSI0922	0.880	0.201	11.000	8.313	2.236	0.838	0.880	0.047	0.868
CmSI0930	0.794	0.302	9.000	4.844	1.773	0.691	0.794	0.130	0.766
CmSI0938	0.751	0.341	8.000	4.021	1.506	0.644	0.751	0.142	0.709
CmSI0396	0.561	0.496	4.000	2.276	0.937	0.627	0.561	−0.118	0.465
CmSI0800	0.800	0.371	10.000	4.995	1.873	0.741	0.800	0.074	0.779
CmSI0881	0.793	0.336	11.000	4.824	1.876	0.445	0.793	0.438	0.767
CmSI0702	0.813	0.277	7.000	5.353	1.787	0.810	0.813	0.004	0.788
CmSI0617	0.665	0.516	7.000	2.986	1.370	0.611	0.665	0.081	0.626
Mean	0.740	0.387	8.294	4.423	1.625	0.640	0.740	0.130	0.705

Note: MAF, minor allele frequency; Na, no. of different alleles; Ne, no. of effective alleles; *I*, Shannon’s information index; Ho, observed heterozygosity; He, expected heterozygosity; and F, fixation index.

**Table 2:** AMOVA analysis of different Japanese chestnut populations.

Source of Variance	Sum of Squares (df)	Sum of Squares (SS)	Mean Square (MS)	Estimated Variance	Variation (%)	*p*
Among Pops	1	57.443	57.443	0.440	7%	<0.01
Within Pops	141	1775.381	4.975	6.305	93%	<0.01
Total	142	1832.824		6.745	100%	

**Table 3:** *t*-test of genetic data of the core and original germplasm.

Germplasm	Collection Number	Total Allele Points	*Na*	*Ne*	*MAF*	*Ho*	*He*	*PIC*	GD
Original germplasm	142	141	8.29	4.423	0.388	0.64	0.74	0.71	0.740
Core germplasm	41	141	7.29	4.236	0.405	0.63	0.73	0.69	0.727
Retention rate%	28.87%	100.00%	87.94%	95.77%	104.38%	98.44%	98.65%	97.18%	97.30%
*t*-value			1.219	0.914	1.693	0.4923	0.335	2.154	0.916
*p*-value			0.332	0.750	0.1098	0.6292	0.739	0.0469	0.739

**Table 4:** Statistical data on the genetic diversity of the 41 core germplasm.

Locus	GD	MAF	*Na*	*Ne*	*I*	*Ho*	*He*	*F*	*PIC*
CmSI0509	0.805	0.232	6.000	5.117	1.673	0.561	0.805	0.303	0.775
CmSI0561	0.438	0.716	4.000	1.779	0.798	0.459	0.438	−0.049	0.387
CmSI0614	0.747	0.438	8.000	3.959	1.672	0.583	0.747	0.220	0.721
CmSI0658	0.715	0.482	8.000	3.508	1.605	0.714	0.715	0.001	0.688
CmSI0735	0.651	0.500	4.000	2.861	1.196	0.659	0.651	−0.012	0.596
CmSI0742	0.664	0.512	7.000	2.978	1.342	0.366	0.664	0.449	0.623
CmSI0853	0.755	0.425	8.000	4.087	1.684	0.775	0.755	−0.026	0.729
CmSI0871	0.858	0.207	11.000	7.063	2.099	0.683	0.858	0.204	0.842
CmSI0883	0.682	0.500	7.000	3.141	1.443	0.567	0.682	0.169	0.645
CmSI0922	0.888	0.159	11.000	8.894	2.275	0.756	0.888	0.148	0.877
CmSI0930	0.814	0.317	9.000	5.379	1.885	0.780	0.814	0.041	0.792
CmSI0938	0.747	0.295	5.000	3.956	1.436	0.564	0.747	0.245	0.702
CmSI0396	0.574	0.500	4.000	2.349	0.980	0.683	0.574	−0.189	0.485
CmSI0800	0.778	0.410	8.000	4.513	1.803	0.744	0.778	0.045	0.758
CmSI0881	0.767	0.392	10.000	4.285	1.776	0.486	0.767	0.365	0.739
CmSI0702	0.804	0.295	7.000	5.104	1.745	0.769	0.804	0.043	0.777
CmSI0617	0.670	0.500	7.000	3.032	1.362	0.611	0.670	0.088	0.627
Mean	0.727	0.405	7.294	4.236	1.575	0.633	0.727	0.120	0.692

**Table 5:** The 41 core germplasm samples.

Number	Cultivars (Lines)	Origin Region	Number	Cultivars (Lines)	Origin Region
1	Youmo	Japan	22	1703	Dalian, Liaoning
2	Fangyangyu	Japan	23	1404	Dalian, Liaoning
3	Liaoli No. 10	Fengcheng, Liaoning	24	Shanda	Republic of Korea
4	08-3	Kuandin, Liaoning	25	Yuguang	Republic of Korea
5	Hemu Shisheng	Fengcheng, Liaoning	26	Dabao	Republic of Korea
6	Guojian	Japan	27	Guangyin	Republic of Korea
7	Kuanyou 9113	Kuandin, Liaoning	28	Danze	Japan
8	Dongshi No. 2	Donggang, Liaoning	29	Wuci	Japan
9	Chuyun	Japan	30	Nonglin No. 8	Japan
10	Gaojiangan	Japan	31	1207	Dalian, Liaoning
11	Liaolizaofeng	Dalian, Liaoning	32	1121	Dalian, Liaoning
12	Liping	Japan	33	Dacheng No. 3	N. Korea
13	Baoyingduguo	Fengcheng, Liaoning	34	1201	Dalian, Liaoning
14	Yinji	Japan	35	Hongqiduguo	Fengcheng, Liaoning
15	Liaodan No. 61	Kuandin, Liaoning	36	1113	Dalian, Liaoning
16	1601	Dalian, Liaoning	37	1120	Dalian, Liaoning
17	Zhubo	Japan	38	1111	Dalian, Liaoning
18	02-6	Kuandin, Liaoning	39	9716	Kuandin, Liaoning
19	0420	Kuandin, Liaoning	40	1066	Dalian, Liaoning
20	Danli No. 3	Japan	41	04-3	Kuandin, Liaoning
21	1502	Dalian, Liaoning			

## Data Availability

The original contributions presented in this study are included in the article/Supplementary Material. Further inquiries can be directed to the corresponding authors.

## Supplementary Materials

The following supporting information can be downloaded at: https://www.mdpi.com/article/10.3390/plants14131998/s1, Table S1: Information sheet of the 200 tested materials; Table S2: The information of 17 SSR markers.

## Abbreviations

The following abbreviations are used in this manuscript:

GBS	genotyping by sequencing
GD	genetic distance
MAF	major allele frequency
PCoA	principal coordinate analysis
PCR	polymerase chain reaction
PIC	polymorphism information content
SNP	single nucleotide polymorphism
SSR	simple sequence repeat
